# Identification of Genes Involved in Resistance to High Exogenous 20-Hydroxyecdysone in *Spodoptera litura*

**DOI:** 10.3390/insects13030297

**Published:** 2022-03-17

**Authors:** Zhijun Dai, Bangyong Sun, Yun Wang, Ze Zhang, Wei Sun

**Affiliations:** School of Life Sciences, Chongqing University, Chongqing 401331, China; 18716433548@163.com (Z.D.); 20152602015@cqu.edu.cn (B.S.); 20162602012@cqu.edu.cn (Y.W.); zezhang@cqu.edu.cn (Z.Z.)

**Keywords:** comparative transcriptome, phytoecdysteroids, degradation, noctuid, evolution

## Abstract

**Simple Summary:**

20-hydroxyecdysone (20E), the most active insect ecdysteroids, is also a major form of phytoecdysteroids in some plants. The phytoecdysteroid from plant is generally considered as defensive weapon to prevent ingestion by phytophagous insects. Conversely, insects also evolved resistance mechanisms to combat the plant defensive system. In this study, we dissected the molecular mechanism to explain how noctuid pest (*Spodoptera litura*) resist high dosage of 20E. Besides, comparative transcriptomic analysis using two noctuid insects (*S. litura* and *Helicoverpa armigera*) also revealed that different species always ultilized various starategies to tolerate ingested hormone.

**Abstract:**

To prevent their ingestion by phytophagous insects, plants produce secondary metabolites as defensive weapons. Conversely, insects need to counter these metabolites to survive. Different species, though they are closely related, can evolve distinct strategies to resist plant-derived factors. However, the mechanism under this high divergence resistance is still unclear at a molecular level. In this study, we focus on how *Spodoptera litura* (Lepidoptera; Noctuidae) detoxifies phytoecdysteroids, a class of metabolites capable of disrupting the normal development of insects. Firstly, we find that the *S. litura* show resistance to artificial foods containing a high level of 20-hydroxyecdysone (20E), the major form of phytoecdysteroids, without any adverse effects on growth and development. Furthermore, a comparative transcriptomic analysis between *S. litura* and another noctuid insect (*Helicoverpa armigera*) was performed. Almost all known ecdysteroid degradation pathways including 3-epimerization, 22-phosphorylation, 22-esterification, and 26-hydroxylation were upregulated in the midgut of 20E treated *S. litura* larvae, whereas only 22-esterification and 26-hydroxylation were enhanced in *H. armigera* larvae. In summary, though both species belong to the Noctuidae family, they evolved two different strategies to tolerate a high dosage of ingested 20E.

## 1. Introduction

Ecdysteroids, the major developmental hormones of insects, have been well known for regulating molting, metamorphosis, and reproduction in insects [1,2]. Besides, ecdysteroids are also widely detected amongst plants. In plants, ecdysteroid analogs are also called phytoecdysteroids which are important compounds of secondary metabolites [3,4,5]. Importantly, the major form of phytoecdysteroids in plants is 20-hydroxyecdysone (20E), which is also the most biologically active ecdysteroid in insects [6]. It is generally believed that the abnormal level of 20E concentration could disrupt the normal life processes of insects [4]. Therefore, phytoecdysteroids are usually considered defense compounds to protect plants against phytophagous insects [7,8]. Indeed, an increase in 20E in an artificial diet stimulated *Bradysia impatiens* and *Popillia japonica* feeding deterrence [9,10]. In addition, orally ingested or injected low levels of phytoecdysteroids could directly induce the death of insects, such as *Bombyx mori* [11]. These phenomena have been demonstrated in some lepidopteran insects [11,12,13,14].

However, certain insects have developed resistance mechanisms to combat the plant defensive system over 350 million years of co-evolution. For example, *Helicoverpa virescens* and *Helicoverpa armigera* can still grow and develop normally without any adverse effects after ingesting high concentrations of 20E (1000 ppm 20E or 50 μg/per larva) [13,15]. In addition, *Lacanobia oleraceae* also exhibited excellent tolerance for the exogenous application of 20E [16]. Some previous studies demonstrated that inactivation pathways of ecdysteroids may contribute to high resistance to 20E, including 3-epimerization, 26-hydroxylation, and 22-fatty-acyl esterification [15,17,18]. The metabolites of inactivation pathways, 3-dehydroecdysone, 20-hydroxyecdysonoic acid, and ecdysteroid-22-acyl esters, all show much less activity than 20E [19,20]. It is important to point out that the inactivation mechanisms in different insects present great variation [21]. However, the molecular mechanism under this variation is still unclear.

*Spodoptera litura* (Lepidoptera: Noctuidae) is a widely distributed crop pest that feeds on about 300 plant species, causing a significant impact on economic crops [22]. As a typical polyphagous insect, *S. litura* shows high tolerance to ingested 20E [23]. However, the high tolerance mechanism of *S. litura* is still unclear. In addition, our previous study demonstrated that the ecdysteroid esterification pathway can confer with cotton bollworm, which also belongs to Noctuidae, to resist a high dosage of exogenous 20E. The data give us an opportunity to investigate how different species adapt to the defense system of their host plants. Here, we firstly surveyed the effect of a high dosage of exogenous 20E on the growth and development of *S. litura* larvae. Thereafter, RNA sequencing was performed to measure the influence of the hormone. Then, we used comparative transcriptomic analysis to identify genes affected by 20E treatment between the two noctuid insects. It is expected that our data would help us understand how insects adapt to the defense system from host plants.

## 2. Materials and Methods

### 2.1. Insects Culture and Treatment

The larvae of *Spodoptera litura*, provided by the Laboratory of Evolution and Functional Genomics, School of Life Sciences of Chongqing University, were reared on an artificial diet in a controlled growth chamber (12-h-light/12-h-dark cycle, 28 °C, 60% humidity). The artificial diet was composed of soybean powder, wheat bran powder, casein, brewer’s yeast, ascorbic acid, methyl p-hydroxybenzoate, sorbic acid, agar, cholesterol, and water.

The artificial diet was cut into 0.25 mg and was mixed with 10 μg, 20 μg, and 50 μg 20E (Chemical Abstracts Service number: 5289-74-7, Sigma-Aldrich, St. Louis, MO, USA; dissolved in 20% ethanol), respectively. The same weight diet with 20% ethanol was used as the control. For ingestion experiments, the day 1 larvae of the sixth instar were starved for 24 h, and then each larva was fed on the artificial diet treated with 20E or ethanol. The larvae that could completely eat the diet within two hours were transferred to the normal artificial diet block. Larvae were weighed at different time points after treatment. Then, some indexes including body weight change trend, body weight gain, time to pupation, and pupation rate were measured.

### 2.2. Total RNA Extraction and RNA Sequencing

In general, the larval midgut is the principal place to detoxify the exogenous 20E. Therefore, the RNA, which was extracted from the midgut collected at 3 h after 20 μg 20E treatment, was subjected to transcriptomic analysis. Each midgut sample was collected from five larvae and ground in liquid nitrogen to a powder. Total RNA was extracted using a TransZoL up Plus RNA Kit according to the manufacturer’s protocol (Beijing TransGen Biotech, Beijing, China). RNA purity was checked using a Nano Photometer spectrophotometer (Implen, Westlake Village, CA, USA). RNA 6000 Nano Assay Kit and Bioanalyzer 2100 (Agilent Technologies, Santa Clara, CA, USA) were implemented to assess RNA integrity. Then, 3 μg of total RNA per sample was used as input material for RNA sequencing. The transcriptome libraries were generated using Illumina TruSeq™ RNA Sample Preparation Kit (Illumina, San Diego, CA, USA) following the manufacturer’s recommendations. RNA-Seq based transcriptome profiling was performed by Biomarker Technologies Corporation (Beijing, China). NovaSeq 6000 platform was applied to generate 150 bp paired-end reads. Each biological sample was repeated two times. The raw data of RNA-seq has been deposited into China National Center for Bioinformation (ID: CRA006208).

### 2.3. Transcriptome Data Analysis

#### 2.3.1. Quality Control, Mapping, and Assembly of Clean Reads

Raw data in the FASTQ format were trimmed using trimmomatic v0.39 with default parameters. FastQC v0.11.9 was performed to calculate the Q20, Q30, and GC content and the sequence duplication level of the clean reads. The reference genome and gene model annotation files were obtained from NCBI (https://www.ncbi.nlm.nih.gov/, 10 December 2019). The clean reads were mapped to the *S. litura* reference genome (GCA_002706865.1) using HISAT2 software v2.2.1 [24]. Stringtie was used to identify known and novel transcripts from the HISAT2 alignment results [24].

#### 2.3.2. Quantification and Differential Expression Analysis of Transcripts

HTSeq was performed to calculate the read counts of each transcript [25]. The expression levels of genes were quantified using TPM (Transcripts Per Million). Differently expressed genes (DEGs) were identified using the DEseq2 R package (release 1.32.0) [26]. A corrected P-value of 0.05 and log2 fold-change (log2FC) of ±1 were set as the threshold for significantly differential expression.

#### 2.3.3. GO and KEGG Enrichment Analyses

To perform GO enrichment analysis, we first performed InterProScan (http://www.ebi.ac.uk/interpro/, v87.0) and BLASTX to obtain the all genes ontology annotation files. The statistical significance of the functional GO enrichment was evaluated using the R package “clusterProfiler” (false discovery rate (FDR) < 0.05). The KOBAS system was used to identify the significantly enriched KEGG pathways [27].

#### 2.3.4. Real-Time PCR

The qRT-PCR was used to determine and verify the expression levels of 13 genes in the midgut. The gene-specific primers were designed based on the specific sequence of the gene and are listed in Supplementary Table S1. Quantitative PCR was performed using a CFX96™ Real-Time PCR Detection System (Bio-Rad, Hercules, CA, USA) with SYBR Green qPCR Mix (Bio-Rad). The cycling parameters were as follows: 30 s at 95 °C, followed by 40 cycles of 95 °C for 10 s, annealing at 52 °C for 30 s. Each sample was tested in triplicate, and the *actin 3* gene was used as the reference gene. Relative gene expression was calculated using the formula R = 2^−ΔΔCt^ [28].

#### 2.3.5. Phylogeny Analysis

*S. litura* Cyp18a1 and Cyp18b1 proteins were used as queries to search their orthologous genes in other insects. Multiple alignments of all identified protein sequences were made by MUSCLE 3.6 [29]. The neighbor-joining method was performed to construct a phylogeny tree in MEGA X [30]. One thousand bootstraps were used to test the phylogeny tree.

## 3. Results

### 3.1. Tolerance of 20E in Spodoptera litura

**Figure 1:**
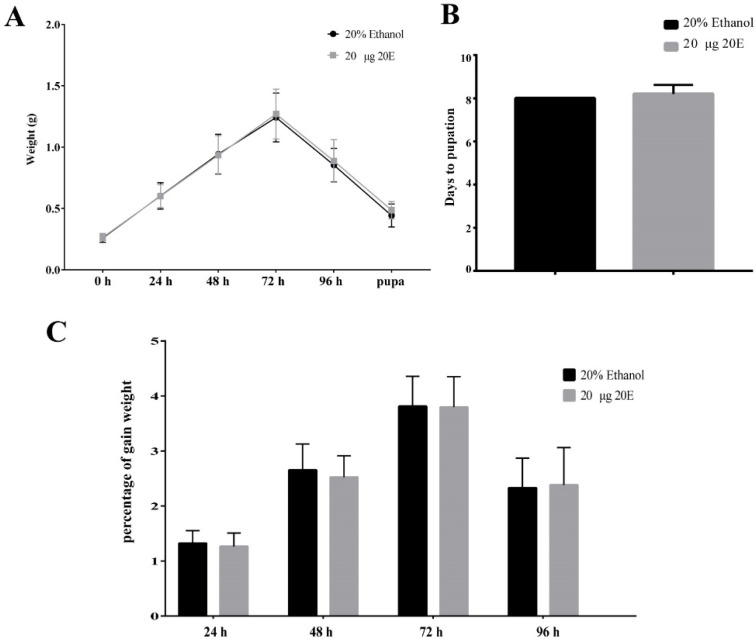
Effects of the ingested 20E (20 μg/larva) on the growth and development of the *S. litura* larvae. (**A**) The average weight of the larvae treated with 20E or 20% ethanol. (**B**) The time to pupation of the larvae after 20E or 20% ethanol treatment. (**C**) Percentage of the gain weight of the larvae treated with 20E or 20% ethanol.

To explore the effect of exogenous 20E on the growth and development of the *S. litura*, we fed the day 1 larvae of the sixth instar with 10 μg, 20 μg, and 50 μg exogenous 20E, respectively. The concentrations of 20E used here are much higher (about 30, 60, and 150 folds) than the top peak of 20E during the metamorphosis stage of the *S. litura* [31]. The results of body weight change trend, time to pupation, and pupation rate showed that all the tested concentrations of 20E did not affect the growth and development of *S. litura* larvae (Figure 1A,B and Figure S1A,B). The treated larvae appeared to experience similar fluctuations in body weight. In addition, the 20E treated larvae also produced normal pupae and adults, the same as the controls. We also calculated the percentage of gain weight (PGW) to avoid material selection errors. The formula was as follows: Percentage of gain weight (PGW) = (Weight gain/The previous day’s weight) × 100%. PGW of larvae also had no significant difference between treatment and control groups (Figure 1C and Figure S1C). Thus, the results revealed *S. litura* larvae have a good tolerance for the high concentration exogenous 20E. Importantly, the normal development of larvae treated 20E also implied that *S. litura* larva may transform or metabolize 20E in some unknown mechanisms.

### 3.2. Transcriptomic Analyses

**Figure 2:**
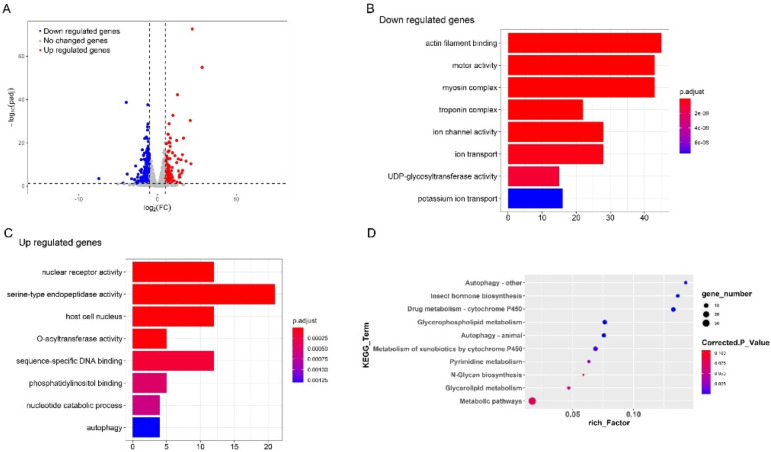
Comparative transcriptome analysis of the larvae midgut treated with 20E or 20% ethanol. (**A**) Volcano plot of the differently expressed genes in the midgut of *S. litura*. The dotted lines indicate thresholds set for regulation (log2 (FC) < −1 and log2 (FC) > 1) and the significance (*t*-test *p*-value < 0.05). Significantly up-regulated genes in 20E treated larvae midgut are indicated in red spots whereas significantly down-regulated genes are marked in blue. (**B**,**C**) Scatterplot of enriched GO terms for up-regulated genes and down-regulated genes, respectively. (**D**) KEGG pathway enrichment analysis of the differently expressed genes.

To understand the mechanism to detoxify the high level of exogenous 20E in *S. litura* midgut, we further examined the genes that play important roles in this process by RNA-seq. The information about the sequencing and assembly is shown in Table 1. By comparing the expression levels of genes between the control and 20E treated larvae, a total of 569 genes were identified to be differentially expressed (Figure 2A). Among these genes, 291 genes were significantly down-regulated after exogenous 20E treatment, and 278 genes were increased. GO enrichment analysis was performed for the differently expressed genes (DEGs). The functional GO terms of down-regulated genes were mainly enriched in transmembrane transport and carboxylate hydrolase activity (Figure 2B), indicating exogenous 20E affected the transmembrane transportability of the midgut. For the up-regulated genes, the top enriched terms are “steroid hormone receptor activity” and “steroid hormone-mediated signaling pathway”, suggesting 20E serves as an important steroid hormone and may change gene expression level in midgut through signal transduction (Figure 2C). As the important steroid hormone, 20E could elicit its signal transduction and affect the expression level of targets. Here, we identified nine transcription factors in the 20E up-regulated gene dataset. Two of them are known 20E response genes, such as ecdysone receptor (EcR) and hormone receptor 3 (Hr3) (Table 2). Hr3 is considered a central regulator in 20E-driven developmental transitions and showed the largest expressional change after 20E treatment. The enriched KEGG pathways of metabolism and cytochrome P450 (Cyp) further indicated that *S. litura* larva may promote the metabolism and detoxification system to tolerate exogenous 20E (Figure 2D).

### 3.3. Identification of Genes Related to the Metabolism of 20E

To identify the candidate genes related to the high ecdysteroids resistance of the *S. litura* larva, we focused on the genes with dramatic expression changes (|log2FC| > 2 and tpm > 5) (Table 3). A total of 32 genes were identified as candidate genes, and 14 were significantly up-expressed genes in the 20E treatment samples. Interestingly, among them (14 genes), we identified 5 enzymes involved in the catabolism of ecdysteroids, including cytochrome P450 18a1, cytochrome P450 18b1, ecdysteroids oxidase, ecdysteroid 22-kinase, and long-chain fatty-acid–CoA ligase. Cyp18b1 is the most differentially expressed gene after the administration of 20E. This gene is paralogous of Cyp18a1, which encodes ecdysteroid 26-hydroxylase. Previous studies demonstrated that *Cyp18a1* could effectively reduce the 20E titers and induce ecdysteroid inactivation through 26-hydroxylation [17]. Ecdysteroid oxidase (*EO*) catalyzes the conversion of 20E to 3-dehydroecdysone (3DE), which has less activity than ecdysone [32,33]. Ecdysteroid 22-kinase (*Ec22K*) plays a key role in the conversion of free ecdysteroids into physiologically inactive ecdysteroid 22-phosphates [34]. For long-chain-fatty-acid–CoA ligase (Long-FACL), the enzyme catalyzes the formation of fatty acyl-CoA, which is involved in various metabolic and regulatory processes. More importantly, our previous study proved the product of Long-FACL could be used as an acyl-group donor to enhance the ecdysteroid-22-O-acyltransferase activity of sterol O-acyltransferase (SATF) [35]. In summary, *S. litura* larvae could utilize almost all known ecdysteroid degradation related enzymes to detoxify the exogenous 20E.

Based on the transcriptomic analyses, we found some genes involved in ecdysone metabolism were activated by exogenous 20E. Ecdysone usually regulates downstream effector genes by activating transcription factors rather than directly interacting with these genes [2]. To further explore the relationship between 20E and ecdysone metabolic genes, we surveyed the 5′ upstream of the ecdysone metabolic genes (*Cyp18a1*, *EO* and *Ec22K*) and identified several 20E related cis-regulatory sites by bioinformatics prediction. Interestingly, these TFs were significantly up-regulated in 20E treated larval midgut, such as EcR/ultraspiracle (USP) (Table 2). The result further indicated these ecdysone metabolic genes were 20E inducible genes.

### 3.4. Evolution of Cyp18a1 and Cyp18b1 Genes in Insects

**Figure 3:**
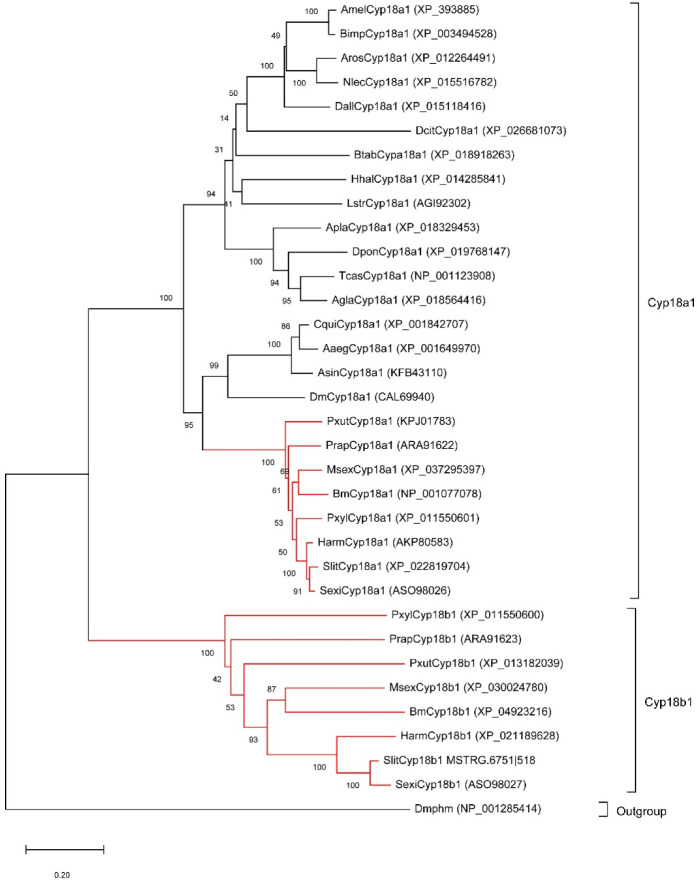
Neighbor-joining tree of insect Cyp18 proteins. The red branches mean the proteins from lepidopteran insects. The *Drosophila melanogaster* Phantom (Dmphm), which also belongs to the Cytochromes P450 superfamily, was used as the outgroup.

As the important 20E metabolic enzymes, we tracked the evolution of Cyp18a1 and Cyp18b1 in insects. Firstly, we identified Cyp18a1 and Cyp18b1 proteins in other insects belonging to Lepidoptera, Diptera, Coleoptera, Hymenoptera, and Hemiptera. Interestingly, Cyp18a1 was identified in all species, whereas Cyp18b1 was only present in Lepidopteran insects. The phylogenetic tree showed all Cyp18a1 proteins were clustered together and then grouped with Cyp18b1 clade (Figure 3). This result indicated that Cyp18b1 was a lepidopteran-specific enzyme that originated after the split of Lepidoptera and other insects.

### 3.5. Quantitative Real-Time PCR

**Figure 4:**
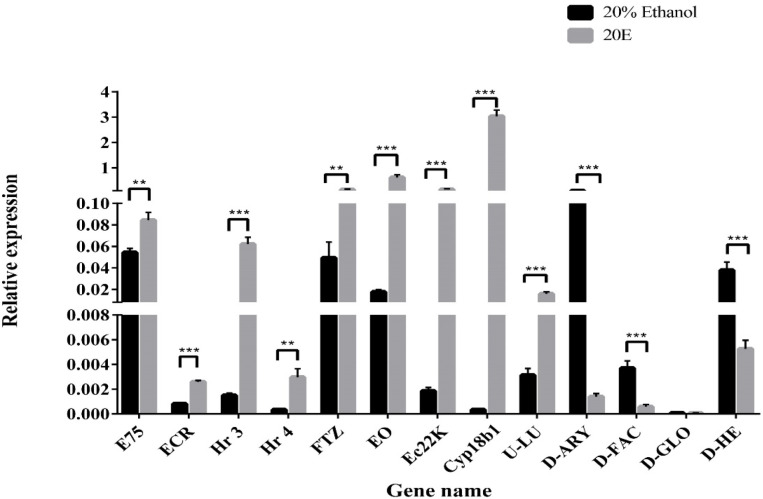
Quantitative real-time PCR validation of the differently expressed genes. Relative gene expression was calculated using the formula 2^−ΔΔCt^ and normalized against HQ012003.2 (GAPDH). Error bars represent the mean ± SD, from one experiment run in triplicate. Statistical significance was analyzed with unpaired Student’s *t*-test (** *p* < 0.01, and *** *p* < 0.001).

Quantitative real-time PCR (qPCR) was performed to validate the RNA-seq data. Overall, 13 DGEs were selected from 20E treatment versus control transcriptome data, including 5 TFs, 3 genes associated with 20E metabolism, 2 AMPs, and 3 other genes (Figure 4). TFs (*E75, EcR, Hr3, Hr4,* and *FTZ*), *Cyp18b1*, *EO,* and *Ec22K* showed up-regulated expression after 20E treatment. This result further supports that *S. litura* larva can tolerate high concentrations of 20E through activating the expression of ecdysone metabolic genes. In contrast, the transcriptional levels of *hemolin* and *gloverin* significantly decreased in tested groups, suggesting 20E serves as a suppressor to affect AMP mRNA expression. In addition, the changing trend of three other genes was like the results obtained from the transcriptome data.

### 3.6. Comparative Transcriptomic Analysis between S. litura and H. armigera

**Figure 5:**
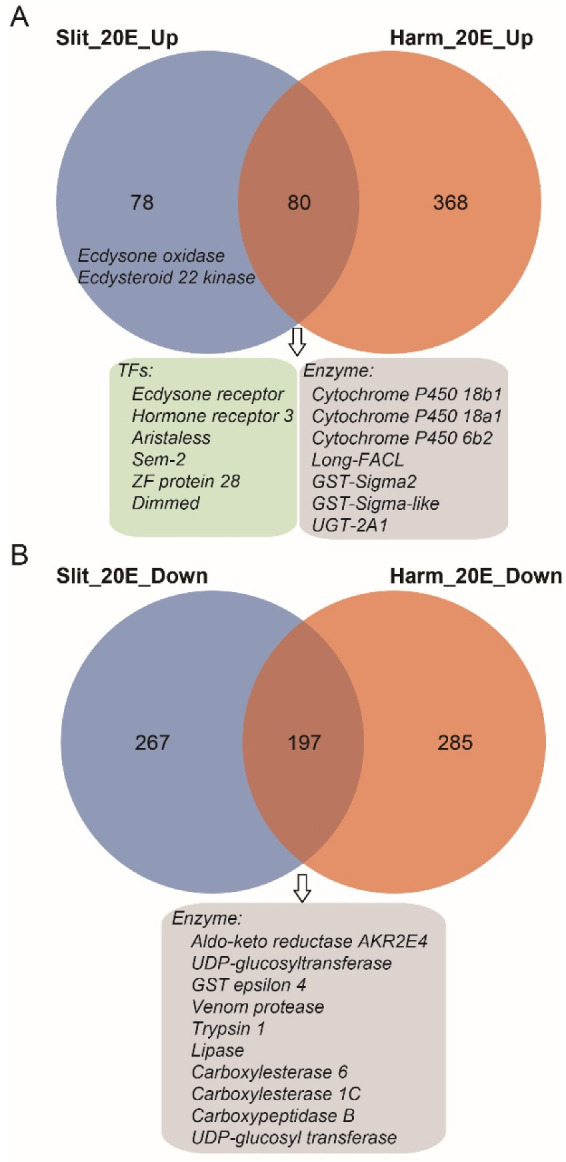
Venn diagram of differentially expressed genes (DEGs) between *S. litura* and *H. armigera*. (**A**) The overlaps of significantly up-regulated gene dataset. (**B**) The overlaps of significantly down-regulated gene dataset. The blue and orange circles represent the gene dataset of *S. litura* and *H. armigera*, respectively. Some genes from overlap gene sets were listed in box.

*S. litura* and *H. armigera* belong to noctuid insects, and both show high resistance to phytoecdysteroids [35]. Our previous study indicated the ecdysteroid esterification pathway was the main process to detoxify exogenous 20E for *H. armigera* [35]. To compare genes affected by 20E treatment between *S. litura* and *H. armigera*, we performed comparative transcriptomic analysis using RNA-seq data from this study and our previous published paper [35]. For 20E up-regulated genes, *S. litura* and *H. armigera* shared 50.6%% (80/158) genes (Figure 5A). In this intersection dataset, several 20E response transcription factors are involved, such as *EcR* and *Hr3*, indicating that similar signal transduction pathways are induced in both insects. Besides, the transcript levels of ecdysteroid 26-hydroxylation related genes (*Cyp18a1* and *Cyp18b1*), ecdysteroid esterification related genes (Long-FACL), and several detoxifying related genes (*Cyp6b2*, GST-sigma, UGT2A1) are also elevated in *S. litura* and *H. armigera*. Interestingly, the ecdysteroid-3 epimerization pathway (ecdysone oxidase) and ecdysteroid-22 phosphate pathway (Ecdysteroid 22 kinase) were only detected in the *S. litura* dataset. Though both *S. litura* and *H. armigera* larva could tolerate high concentrations of 20E, they evolved different strategies to adapt to and combat the phytoecdysteroids of their host plants.

For the 20E down-regulated genes, we identified 197 genes that were shared with *S. litura* and *H. armigera* (Figure 5B). Among them, some digestive enzymes (trypsin, lipase, and carboxypeptidase) decreased after the 20E treatment. It seems that 20E could affect the normal nutrition conversion of larvae. However, the feeding test shown above indicates that the steroid hormone had no negative effects on the growth and developmental process of the larvae (Figure 1). Indeed, in both insect larvae, we detected most of the other digestive enzyme genes, including carbohydrases (β-glucosidase, α-amylase, trehalase, α-glucosidase, and β-galactosidase) and aminopeptidases, have no significant expression change after the hormone treatment.

## 4. Discussion

Most insects feed on plants [36]. As sessile organisms, plants do not effectively escape attacks from insects, so they must employ other strategies to defend themselves. The important insect hormone 20-hydroxyecdysone can regulate multiple life processes, from molting to metamorphosis [1,2]. Interestingly, 20-hydroxyecdysone also represents a major plant phytoecdysteroid that mimics insect hormones and interferes with insect molting [8]. Therefore, it is broadly accepted that the phytoecdysteroid serves as a defender to resist herbivores [6,37,38]. However, some insects have also developed strategies to overcome the defensive system during their long coexistence with plants [13,15,35]. To better understand how insects adapt to the phytoecdysteroid of their host plants, we demonstrated how the physiology and gene expression levels change after 20E treatment in *S. litura* larvae.

In this study, we found that the growth and development of *S. litura* larvae were not affected significantly by high concentrations of ingested exogenous 20E (up to 50 μg/larva). This phenomenon is similar to its closely related species *Spodoptera littoralis* [39]. In the feces and gut of *S. littoralis* treated with 20E, the authors identified ecdysteroid 22–fatty acyl esters and 20-hydroxyecdysonoic acid, indicating *S. littoralis* tolerate 20E by degradation. The midgut is an important organ for insects to detoxify the plant allelochemicals [40]. Thus, the identification of 20E induced genes in the midgut may help us to further understand the molecular mechanism of ecdysteroid tolerance in *S. litura* larvae. According to the transcriptome data, we found some candidate genes involved in ecdysteroid transformation and metabolism in different ways. For example, *Cyp18b1* showed the greatest difference in expression level after 20E treatment. *Cyp18b1* and its paralogous *Cyp18a1* belong to the cytochrome P450s family, which has been shown to play key roles in the metabolism of insecticides and host plant chemicals [41]. *Cyp18a1* is a conserved gene present in most arthropods. However, *Cyp18b1* was only detected in lepidopteran species, and it was generated by lineage-specific gene duplication after the Lepidoptera-other taxa split. It should be noted that the function of *Cyp18b1* is still unknown [17]. We also found Long-FACL was up-regulated more than fourfold after 20E treatment. Duan et al. [35] suggested Long-FACL can help 20E esterase to initiate esterification of exogenous ecdysterone in cotton bollworm midgut. In addition, the fold changes in *EO* and *ECK* expression levels were also dramatic, suggesting 3-epimerization and phosphorylation pathways may play part roles in the inactivation of ingested 20E. Compared with its related species, *H. armigera*, which mainly executes ecdysteroid 26-hydroxylation and 22-esterification pathways to detoxify the exogenous 20E, *S. litura* may employ more strategies to inactivate ingested hormone, such as 3-epimerization and phosphorylation pathway.

## 5. Conclusions

In conclusion, our and previous studies have shown some truly polyphagous species, including *S. litura* and *H. armigera*, feed on the high concentration of ecdysteroid without any detrimental effects owing to their highly efficient hormone degradation pathways. However, different species, even though they are closely related, may evolve various mechanisms to adapt to and combat the secondary metabolites of their host plants.

## Figures and Tables

**Table 1:** Summary of the sequence assembly after Illumina sequencing.

Sample ID	Clean Reads	Clean Bases (Gb)	Read Length (Base Pairs)	Mapping Rate (%)
20E-3H-1	46,738,656	6.33	150 × 2	93.59
20E-3H-2	53,305,123	7.18	150 × 2	93.54
20E-3H-3	47,689,017	6.41	150 × 2	93.06
C-3H-1	44,390,170	6.11	150 × 2	93.58
C-3H-2	43,164,171	5.97	150 × 2	93.75
C-3H3	37,552,588	6.02	150 × 2	93.50

Gb: Giga base.

**Table 2:** The expression levels of transcription factors in midgut of *S. litura*.

Up/Down	Gene ID	Annotation	Log2 Fold Change	Padj	AVG. TPM-20E	AVG. TPM-Control
Up-regulated	XM_022961202.1	Hr3	5.679	1.497 × 10^−55^	16.042	0.300
	XM_022963764.1	EcR	1.569	2.48 × 10^−66^	8.574	2.889
	XM_022965256.1	Aristaless	1.216	0.02872259	7.514	3.099
	XM_022959627.1	Homeodomain	1.104	1.89 × 10^−5^	8.116	3.623
	XM_022968195.1	Zf-OZF-like	1.060	0.02268665	1.808	0.786
	XM_022958318.1	Sem-2	1.052	3.03 × 10^−12^	10.591	4.894
	XM_022979342.1	Dimmed	1.256	0.02270017	2.060	0.841
	XM_022963022.1	Zf-708	1.379	0.00538809	2.282	0.853
	XM_022979059.1	Zf-28	1.053	0.00389892	2.278	1.050
Down-regulated	XM_022967399.1	Zf-CCHC	−1.012	0.03628638	4.074	7.855
	XM_022973385.1	Scr	−1.452	0.00020233	1.996	5.251
	XM_022970658.1	Homeobox (Ubx)	−2.940	0.00978845	0.238	1.727

**Table 3:** The dramatically changed genes in the gut of *S. litura*.

Up/Down	Gene ID	Annotation	Log2 (FC)	Padj	AVG. TPM-20E	AVG. TPM-Control
Up-regulated	XM_022964033.1	cytochrome P450 18b1	16.09088122	3.017 × 10^−38^	362.9826184	0.001666667
	XM_022961202.1	nuclear hormone receptor HR3	5.679311653	1.497 × 10^−55^	16.04207906	0.300014713
	XM_022967830.1	ecdysteroid 22-kinase	4.420986928	2.933 × 10^−73^	47.62505078	2.117049013
	XM_022970997.1	chymotrypsin-1-like	4.249346261	4.838 × 10^−11^	71.44234406	3.59105572
	XM_022970711.1	brachyurin-like	4.164301133	5.193 × 10^−31^	70.37707131	3.792676227
	XM_022976191.1	glucose 1-dehydrogenase	3.171787135	3.226 × 10^−15^	23.33644902	2.514047173
	XM_022958830.1	trypsin, alkaline B-like	3.084927024	9.105 × 10^−7^	89.59084232	10.28438286
	XM_022963936.1	cytochrome P450 18a1	3.000918859	7.327 × 10^−13^	41.77379346	5.003354125
	XM_022971323.1	transmembrane protease serine 9	2.644002489	3.039 × 10^−13^	85.16251423	13.1268597
	XM_022975357.1	ecdysone oxidase	2.553535544	6.372 × 10^−43^	392.5159453	63.17630486
	XM_022968019.1	zinc carboxypeptidase	2.463604169	0.013725634	60.95529246	11.00998255
	XM_022958914.1	nose resistant to fluoxetine protein 6	2.444579022	7.867 × 10^−22^	27.29573632	4.718531976
	XM_022965887.1	trypsin CFT-1	2.217237123	7.889 × 10^−15^	29.98568016	6.139305721
	XM_022966766.1	long-chain-fatty-acid--CoA ligase 5	2.109054146	0.002842622	11.62949936	2.552352744
Down-regulated	XM_022978278.1	uncharacterized	−7.465534116	0.000319546	0.631825112	107.7258629
	XM_022970500.1	synaptic vesicle glycoprotein 2C	−4.356672681	0.036047594	0.470264335	9.747893176
	XM_022964864.1	cuticle protein	−3.975394963	2.19 × 10^−39^	0.525535497	7.803673186
	XM_022975734.1	alpha-tocopherol transfer protein	−3.287976938	0.000414331	1.241149402	11.69727807
	XM_022974716.1	pollen-specific leucine-rich repeat extensin	−2.914132347	0.001175939	5.15499562	37.57454181
	XM_022967410.1	cytochrome P450 6B6	−2.775673993	4.75 × 10^−10^	2.705363121	17.22727217
	XM_022975322.1	D-erythronate dehydrogenase	−2.754762003	0.030014009	1.030915417	6.923361775
	XM_022960877.1	lambda-crystallin	−2.673081084	0.038952194	2.545470213	16.26949459
	XM_022981956.1	inorganic phosphate cotransporter	−2.628303121	0.038441826	3.331505842	20.27546975
	XM_022969882.1	flexible cuticle protein	−2.534897839	7.23 × 10^−5^	1.535841026	8.539128344
	XM_022965868.1	monocarboxylate transporter	−2.521292082	0.004046914	2.442314923	13.65468239
	XM_022979094.1	glycine receptor subunit alphaZ1	−2.493651077	0.011281738	1.428065128	7.783297021
	XM_022963349.1	hemolin-like	−2.461582343	3.44 × 10^−6^	3.004833103	15.36012743
	XM_022976208.1	luciferin 4-monooxygenase	−2.19624467	6.53 × 10^−8^	5.932415617	25.83345994
	XM_022968793.1	synaptic vesicle glycoprotein	−2.189341324	0.029815969	65.75122659	297.0126624
	XM_022967111.1	uncharacterized	−2.148207278	0.019741741	10.53439059	45.93050349
	XM_022964438.1	ester hydrolase C11orf54	−2.026208816	0.042975189	3.869685265	15.55821047
	XM_022961970.1	NPC2 homolog	−2.026189718	1.97 × 10^−8^	8.573871211	33.40835245

## Data Availability

Data can be provided on request from the lead author.

## Supplementary Materials

The following are available online at https://www.mdpi.com/article/10.3390/insects13030297/s1. Figure S1: Effects of the ingested 20E (10 μg/larva and 50 μg/larva) on the growth and development of the *S. litura* larvae. (A) The average weight of the larvae treated with 20E or 20% ethanol. (B) The time to pupation of the larvae after 20E or 20% ethanol treatment. (C) Percentage of the gain weight of the larvae treated with 20E or 20% ethanol. Table S1: qPCR primers
