# Supplementary material for: Identification of Genes Involved in Resistance to High Exogenous 20-Hydroxyecdysone in Spodoptera litura
Source: Insects. 2022 Mar 17;13(3):297. doi: 10.3390/insects13030297 (PMC8955939; doi:10.3390/insects13030297)
Supplement: Supplementary file 1 [file insects-13-00297-s001.zip › insects-1490485-supplementary.pdf]

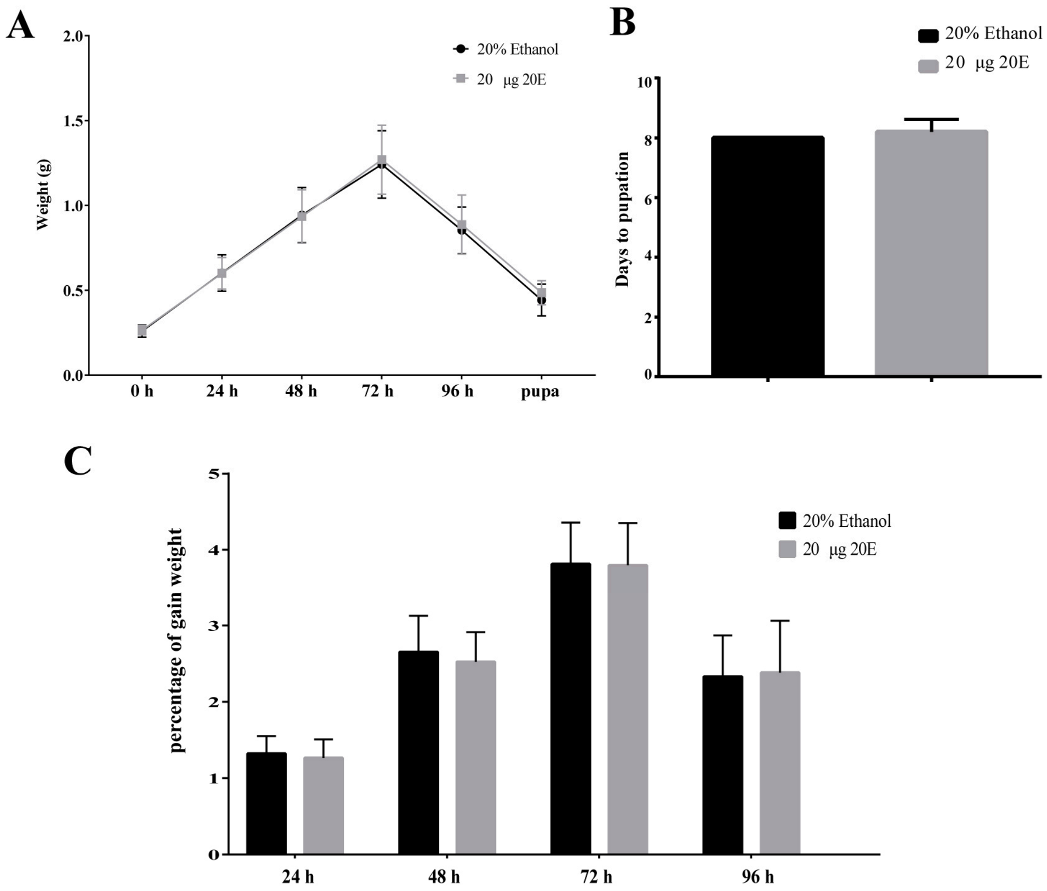

Figure S1. Effects of the ingested 20E (10 μg/larva and 50 μg/larva) on the growth and development of the S. litura larvae. (**A**) The average weight of the larvae treated with 20E or 20% ethanol. (**B**) The time to pupation of the larvae after 20E or 20% ethanol treatment. (**C**) Percentage of the gain weight of the larvae treated with 20E or 20% ethanol.

Table S1 qPCR primers

Gene name	Gene ID	Forward primer	Reverse primer
D-ARY	XM_022966958.1	TGGCAAGGACTATGACAT	TACAGCAGCAAGAACTCT
D-FAC	XM_022973512.1	CCTCAATGTGCTCGTTAA	AACAATAGACAAGTGATTCCT
D-GLO	XM_022981636.1	ACCTTGTGAGAATAACTACCTAGC	AAGCAGCGAAGCAGAGAA
D-HE	XM_022963349.1	CTAAGGATGGAGAGTTGAC	GCAGTAGAACATAGTGACAT
CYP	XM_022964033.1	GAGAAGATATTCCAGGTT	GAGATATGCTTCAGGTAA
HR3	XM_022961202.1	TGAAACTGACGCAAGATG	CGTACAATACTTGGTCACGGTTCAC
ECK	XM_022967830.1	AATTAGAGCAGTCGGTAA	CTATCCAGTCGTAGTAGTAA
EO	XM_022975357.1	GTGTATTGTGGTAGTAAC	GAATAATAGGAAGATTGAATG
HR4	XM_022965367.1	GTCAACATCACAGCATTAG	TAGTTCAGGAGAGTTAGGT
U-LUC	XM_022976208.1	TCAATAAGTATAAGCCAGTATCAA	AGCAGGAGAAGTCACATT
E75	XM_022963581.1	GCTGAAACGCACCGACATCATCCAA	GACTTCTTGGACAGGTTCAGGGGCG
FTZ	XM_022976556.1	GCGACCAAACCGACACTA	CGTTAGCAGCCCGTAGTG
ECR	XM_022963764.1	AGTAGCTCGGCGGTATGA	CTGCCTTGCGGTAGTTGT
Reference gene	HQ012003.2	GGTGCCCAGCAGAACAT	GCAACAGGAACACGGAAA
